# Cross-cultural differences in crossmodal correspondences between basic tastes and visual features

**DOI:** 10.3389/fpsyg.2014.01365

**Published:** 2014-12-08

**Authors:** Xiaoang Wan, Andy T. Woods, Jasper J. F. van den Bosch, Kirsten J. McKenzie, Carlos Velasco, Charles Spence

**Affiliations:** ^1^Department of Psychology, School of Social Sciences, Tsinghua UniversityBeijing, China; ^2^Xperiment, LausanneSwitzerland; ^3^Institute for Learning and Brain Sciences, University of WashingtonSeattle, WA, USA; ^4^School of Psychology, University of Nottingham Malaysia CampusSemenyih, Malaysia; ^5^Crossmodal Research Laboratory, Department of Experimental Psychology, University of OxfordOxford, UK

**Keywords:** crossmodal correspondence, taste/flavor, culture, color, shape, texture

## Abstract

We report a cross-cultural study designed to investigate crossmodal correspondences between a variety of visual features (11 colors, 15 shapes, and 2 textures) and the five basic taste terms (bitter, salty, sour, sweet, and umami). A total of 452 participants from China, India, Malaysia, and the USA viewed color patches, shapes, and textures online and had to choose the taste term that best matched the image and then rate their confidence in their choice. Across the four groups of participants, the results revealed a number of crossmodal correspondences between certain colors/shapes and bitter, sour, and sweet tastes. Crossmodal correspondences were also documented between the color white and smooth/rough textures on the one hand and the salt taste on the other. Cross-cultural differences were observed in the correspondences between certain colors, shapes, and one of the textures and the taste terms. The taste-patterns shown by the participants from the four countries tested in the present study are quite different from one another, and these differences cannot easily be attributed merely to whether a country is Eastern or Western. These findings therefore highlight the impact of cultural background on crossmodal correspondences. As such, they raise a number of interesting questions regarding the neural mechanisms underlying crossmodal correspondences.

## INTRODUCTION

‘Crossmodal correspondence’ is one of the terms that have been used to describe the tendency that people have to associate certain features, or stimuli, across the senses (Spence, 2011; Spence and Deroy, 2013). For example, one of the best known correspondences between sound and shape is the Bouba–Kiki effect. People tend to associate the words “Bouba” (soft sound) and “Kiki” (sharp sound) with rounded and angular shapes, respectively (Köhler, 1929; Marks, 1978; Ramachandran and Hubbard, 2001; Bremner et al., 2013). Even though the majority of the research on crossmodal correspondences that has been conducted to date has focused on correspondences between audition and vision (see Parise and Spence, 2013, for a review), there is also a growing interest in examining and documenting other crossmodal correspondences across different senses (see Spence and Deroy, 2014, for a review).

Spence (2011) distinguished between three different types of crossmodal correspondences, including, (1) structural correspondences which are thought to result from commonalities in the way in which different kinds of sensory information are coded neurally, (2) statistical correspondences, which pick-up on the repeated co-exposure of pairs of stimuli or correlated dimensions of experience in daily life, and (3) the linguistic correspondences that are rooted in language. Both statistical and linguistic correspondences may depend on people’s cultural background and on context, thus leading to possible cross-cultural differences. What is more, a fourth type of crossmodal correspondence might be based on an individual’s affective response to stimuli that they want to pair together (Deroy et al., 2013; Palmer et al., 2013). Yet, it should be noted that the different types of correspondences are not mutually exclusive. For example, people may learn the statistical regularities and develop semantic and/or structural correspondences over the short or long term (e.g., Ernst, 2007; Parise and Spence, 2013).

Previous studies have revealed that people match basic tastes with a host of other non-gustatory stimuli/dimensions, such as colors (see Spence et al., 2010, for a review), and shapes (Deroy and Valentin, 2011; Spence and Gallace, 2011; Spence, 2012; Spence and Ngo, 2012; Spence and Deroy, 2014). Given the profound cultural differences in terms of people’s food preferences and consumption behaviors (e.g., Kittler and Sucher, 2007), it would seem reasonable to expect that there would also be significant cross-cultural differences in the visual-taste/flavor associations they express (cf. Bremner et al., 2013). As a case in point, a growing number of studies have recently started to examine cross-cultural differences in the way in which people match gustatory with non-gustatory information, and investigated color–odor associations (e.g., Levitan et al., 2014), color–flavor associations (e.g., Shankar et al., 2010; Velasco et al., 2014; Wan et al., 2014, 2015), and shape-flavor associations (Bremner et al., 2013; Woods et al., 2013). For example, Western participants associate carbonated water with angular shapes, and still water with rounded shapes (Gallace et al., 2011; Ngo et al., 2012), whereas members of the Himba tribe from rural Namibia do not show any such effect (Bremner et al., 2013). Or, to take another example, when asked to match shapes with chocolate having 30, 70, or 90% cocoa content, Westerners tend to associate flavors that are more bitter with more angular shapes (Ngo et al., 2011), whereas the Himba showed the opposite pattern or results, aligning less bitter flavor with more angular shapes (Bremner et al., 2013).

However, a range of cross-cultural similarities have also been documented (e.g., Piqueras-Fiszman et al., 2012; Ngo et al., 2013; Wan et al., 2015). For example, Ngo et al. (2013) assessed crossmodal correspondences between the taste/flavor of fruit juices and various visual attributes in two different cultures. British and Colombian participants were found to associate sweeter fruit juices with rounder shapes while associating sourer-tasting fruit juices with more angular abstract shapes instead. It would seem likely that future research would reveal both culture-specific and universally shared correspondences, but there is, undoubtedly, a need to understand and define the extent to which correspondences are shared (or not) across cultures.

Importantly, though, none of the above-mentioned studies has thoroughly examined cross-cultural differences in the crossmodal associations between visual features, such as color, shape, and texture^1^ and taste terms, such as bitter, salty, sour, sweet, and umami^2^. In the present study, participants performed a color-taste matching task, a shape-taste matching task, and a texture-matching task, which was similar to the matching task introduced by O’Mahony (1983). He had his participants match the four basic taste terms (bitter, salty, sour, and sweet) with 12 colors (black, blue, brown, gold, green, gray, orange, red, silver, violet, white, and yellow), 7 days of the week, and seven states of the USA. As summarized in **Table 1**, the results revealed some crossmodal associations between red and sweet, yellow and sour, white and salty, as well as green/black and bitter.

**Table 1:** The color-taste associations observed in different studies, for the commonly tested colors.

	O’Mahony (1983)	Tomasik-Krótki and Strojny (2008)	Koch and Koch (2003)	The present study
Number of participants	51	519	45	452
Origin of participants	California, USA	17 countries/areas	Oregon, USA	4 countries
Black	Bitter			Bitter
Blue		Salty		
Green	Bitter	Sour	Sour	Sour
Orange		Sweet	Sweet	
Pink	–	–	–	Sweet
Red	Sweet	Sweet	Sweet	
Violet		Bitter/Umami		–
White	Salty		Salty	Salty
Yellow	Sour	Sour	Sour	

*Note: – denotes that this color was not tested in this study.*

In related research, Tomasik-Krótki and Strojny (2008) had their participants link the five basic taste terms, including umami with a selection of seven colors (blue, green, greenish blue, orange, red, violet, and yellow). As summarized in **Table 1**, the results revealed some crossmodal associations between red/orange and sweet, yellow/green with sour, blue with salty, and violet with bitter/umami. Interestingly, there is some consistency between the results of these two studies (red-sweet and yellow-sour associations, for example), but also discrepancies, such as the specific taste that was associated with color blue and green.

By contrast, Koch and Koch (2003) used a different task in order to examine the crossmodal correspondences between the four basic tastes (as well as four other tastes associated with soft drinks) and eight colors (black, blue, brown, green, orange, purple, red, and yellow). They had their participants rate the degree at which a given color is related to a certain color on the 10-point scale, with higher scores indicating positive associations and lower scores indicating negative associations. As summarized in **Table 1**, the results revealed crossmodal associations between red/orange and sweet, green/yellow and sour, as well as white and salty.

Despite Tomasik-Krótki and Strojny’s (2008) study not addressing cross-cultural differences, it should be noted that their participants came from more than a dozen countries and regions from all over the world. By contrast, all of the participants in O’Mahony’s (1983) as well as Koch and Koch’s (2003) studies were students of major universities in the USA (mostly likely the Western, Educated, Industrialized, Rich, and Democratic, namely WEIRD; Henrich et al., 2010). Therefore, it is possible that the discrepancy between the results of the three studies might be due to the difference in the cultural background of the participants who were tested.

In the present study, we attempted to address these important questions by testing participants from mainland China, India, Malaysia, and the USA. These particular countries were chosen for the following reasons: The USA is one of the most often mentioned countries when it comes to representing “Western culture” in the field of Cultural Psychology, not to mention standard psychological research (Henrich et al., 2010). By contrast, China, India, and Malaysia are all Asian countries, but they exhibit some dramatic differences in terms of their food culture (e.g., Kittler and Sucher, 2007) which might lead to differences in terms of crossmodal associations between visual attributes and taste/flavor.

## MATERIALS AND METHODS

### PARTICIPANTS

A total of 452 participants took part in this study. 428 of them came from four separate populations, including 144 from mainland China (19.8 ± 1.5 years, ranging from 18 to 30 years; 52 females), 113 from India (31.4 ± 9.4 years, ranging from 19 to 66 years; 46 females), 117 from the USA (30.3 ± 8.4 years, ranging from 18 to 68 years; 31 females), and 54 from Malaysia (20.9 ± 4.0 years, ranging from 17 to 36 years; 45 females)^3^. This study was reviewed and approved by the Central University Research Ethics Committee of the University of Oxford. All of the participants provided informed consent prior to taking part in the study. The Malaysian participants were recruited via email, which contained a link to the online experiment. The North American and Indian participants were recruited from Amazon’s Mechanical Turk, and took part in this study in exchange for a payment of 0.80 US dollars. Through a feature of Mechanical Turk, we specified that only people registered as living in America or India could take part in the study. The Chinese participants were undergraduate students from Tsinghua University, Beijing, China, and they received credit to fulfill the requirement of an introductory psychology course.

### MATERIALS

This study was conducted online using Adobe Flash-based Xperiment software (http://www.xperiment.mobi downloaded on 15/05/13). The participants from India, Malaysia, and the USA completed the task in English, whereas the participants from China did so in Chinese. The text descriptors and images used as stimuli were presented against a gray background (RGB: 217, 217, 217), and each stimulus was tightly fit within a 90 × 90 pixel box. Text descriptors of bitter, salty, sour, umami, and sweet were used for the five taste terms, all of which were in the Times New Roman font size 22.

**FIGURE 1:**
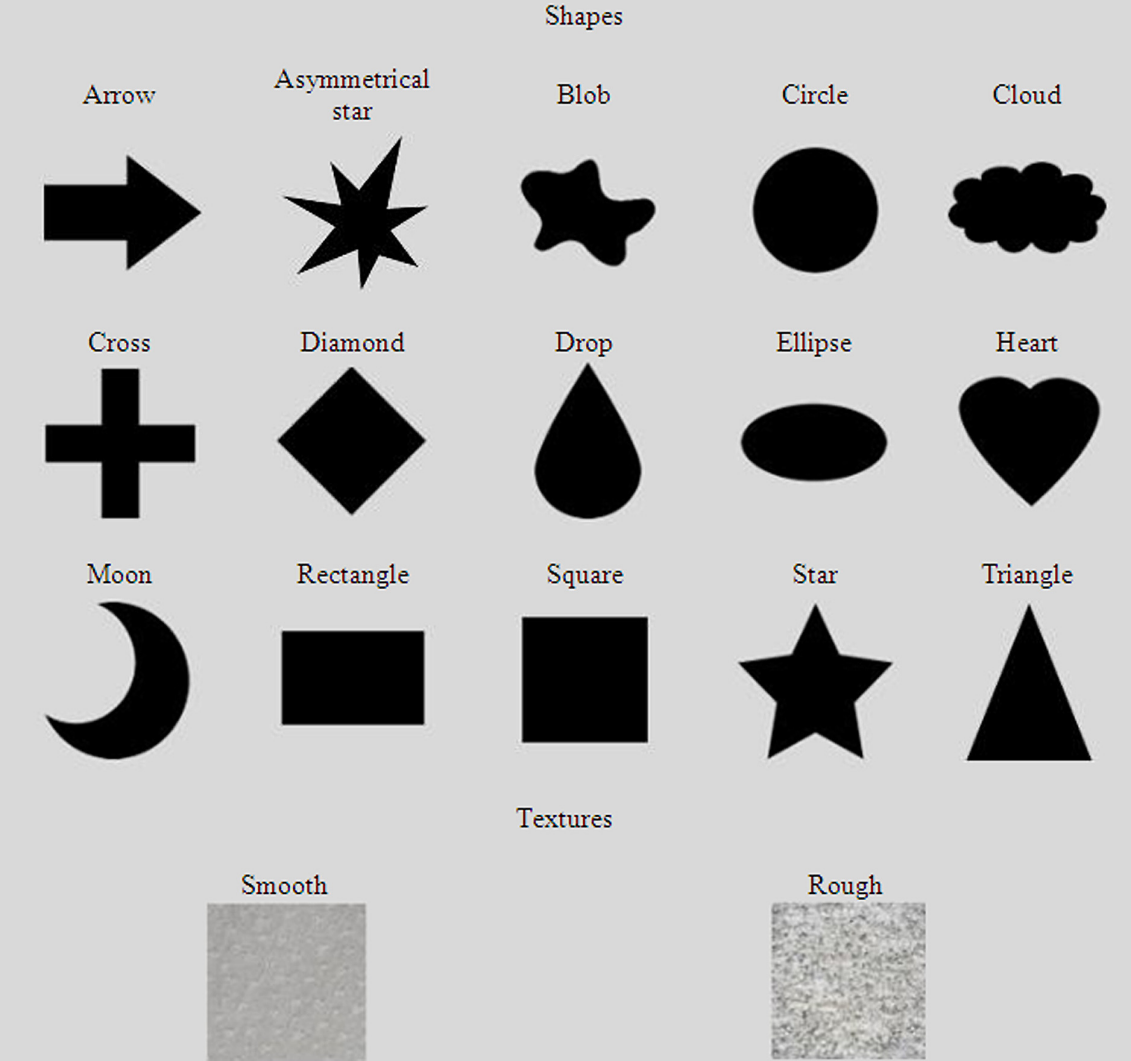
**The 15 shapes and 2 texture patches presented in this study**.

Three types of images were used as the central targets, including the images of shapes, color patches (all presented as a square shape), and texture patches (all in the square shape). Specifically, 11 different color patches were used, including black (RGB: 0, 0, 0), blue (RGB: 0, 0, 255), brown (RGB: 165, 42, 42), green (RGB: 0, 255, 0), gray (RGB: 128, 128, 128), orange (RGB: 255, 165, 0), pink (RGB: 255, 192, 203), purple (RGB: 128, 0, 128), red (RGB: 255, 0, 0), white (RGB: 255, 255, 255), and yellow (RGB: 255, 255, 0). As shown in **Figure 1**, 15 different shapes were presented, all in black, including the arrow, asymmetrical star, blob, circle, cloud, cross, diamond, drop, ellipse, heart, moon, rectangle, square, star, and triangle. Similar to Woods’s et al. (2013) study, two texture patches were used, including both smooth and rough textures (see **Figure 1** for an illustration). Specifically, the smooth texture was visualized as a gray background with sparse granular gray spots, whereas the rough texture was visualized by a gray background with dense granular white spots.

### DESIGN AND PROCEDURE

A within-participants experimental design was used. That is, all of the participants undertook a total of 28 experimental trials (15 shape trials, 11 color trials, and 2 texture trials) which were presented in a random order. At the beginning of the study, the participants were given instructions as to how to complete the study, and completed a single trial in which they were asked to match the taste word ‘umami’ with one of the following words: paper, purple, savory, sweet, watch, yellow. The experiment continued once the participant had correctly answered this question. Participants were directed to the Wikipedia website^4^ for the term “umami” in the case that they wanted a description of this term. The purpose of this practice trial was twofold, as it provided an opportunity for the participants to become familiar with both the click-drag-release task, and the meaning of the term “umami.” After this, the participants were instructed to complete each trial as quickly and as accurately as possible and to click ‘start’ to begin the study.

**FIGURE 2:**
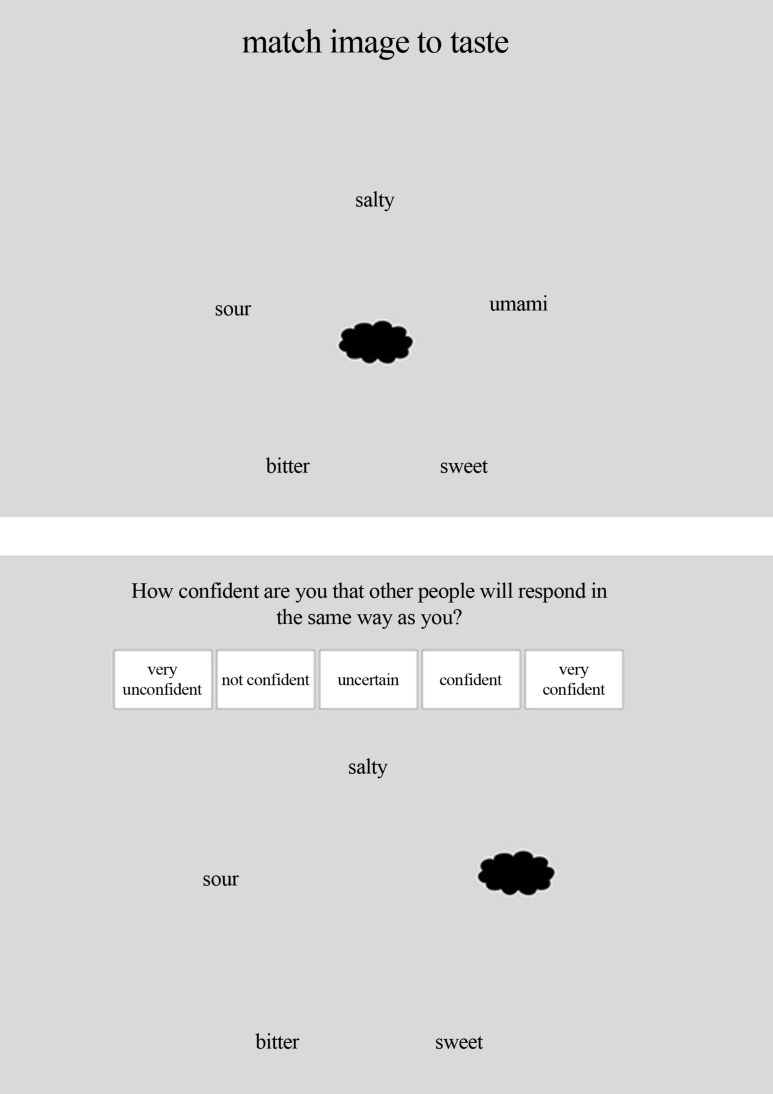
**An illustrations of the screens on which participants were asked to match the central target with one of the taste terms (upper panel) and to indicate how confident they were about their answer (lower panel)**.

During each trial, a central target (e.g., an image of the cloud shape, as shown in **Figure 2**) was presented in the center of the screen, with words representing the five basic tastes arrayed in a pentagon-shape around it. The location of each taste word was varied randomly around the pentagon from trial to trial. The participant’s task involved dragging the target to the word that was associated with it. Specifically, the participants were instructed to (1) place the cursor on the target and press the left mouse button, (2) drag the word by moving the mouse (with the left button still being pressed) until the target overlapped with one of the taste words, and (3) to release the target by releasing the left mouse button. Upon making their response, the participants were asked how confident they were that other people would respond in the same fashion as they had, by indicating on a 5-point horizontal scale with the following options, arranged from left to right: Very unconfident, unconfident, uncertain, confident, and very confident. After they had rated their confidence, a “continue” button appeared on the webpage for them to press in order to initiate the next trial. It took ∼8 min to complete the entire study. After completing the experiment, all of the participants were debriefed as to its purpose.

### DATA RECORDING AND ANALYSES

The key dependent variable in this study was the taste word that the participants chose for each target, from the five possible options. That being said, their confidence rating regarding the consensuality of their answer (Koriat, 2008), i.e., the degree to which the participants felt that others would make the same stimulus pairing, was also included in some of the data analyses.

We first analyzed the chosen tastes across the four groups. We combined the data from the four groups of participants, and performed Chi-square tests with Bonferroni’s correction for multiple testing^5^ to examine whether certain taste(s) were more often associated with a stimulus than other tastes. When the results of these Chi-square tests reached significance, we then performed *post hoc* Chi-square tests to examine whether the taste that was chosen most often was chosen significantly more often than the second most frequently chosen taste.

Next, we analyzed any cross-cultural differences in terms of the tastes that were chosen. We performed log-linear analyses for each stimulus, using taste (bitter, salty, sour, sweet, and umami) × country (China, India, Malaysia, and the USA) as the variables.

After, ‘taste-patterns’ (i.e., the overall pattern of responses across all of the stimuli) were computed for each group of participants, and were weighted by their confidence ratings by being multiplied by the respective coefficient (0 for very unconfident, 0.25 for not confident, 0.5 for uncertain, 0.75 for confident, and 1 for very confident). representational similarity analysis (RSA) was performed using pyRSA (http://github.com/ilogue/pyrsa). The representational dissimilarity matrices (RDMs, see Kriegeskorte et al., 2008) were calculated for each cultural group, in order to compare the crossmodal correspondence between one stimulus and the five tastes (i.e., the taste-pattern) to the taste-pattern of another stimulus. The emerging structure seen in the RDM displays the structure in the taste representations of the stimuli, such as categories. For instance, within one stimulus category, the taste-patterns may be more similar than outside of that category. This would show as a blue (low dissimilarity) block in the RDM. Here, the dissimilarity between two taste-patterns was calculated as 1 minus the correlation coefficient, Pearson’s *r*, between these two patterns. Therefore, each RDM has *n* rows and *n* columns, where *n* represents the number of stimuli that participants were asked to associate with the tastes. After that, we calculated a cross-cultural RDM using the same method in order to compare the crossmodal associations between cultures.

Multidimensional scaling (MDS) was also used in order to display the similarity distances between the stimuli by compressing all of the dimensions into two. In the MDS figure, those stimuli having taste-patterns that were more similar are closer to each other.

## RESULTS

### TASTE-PATTERNS ACROSS THE FOUR GROUPS

**FIGURE 3:**
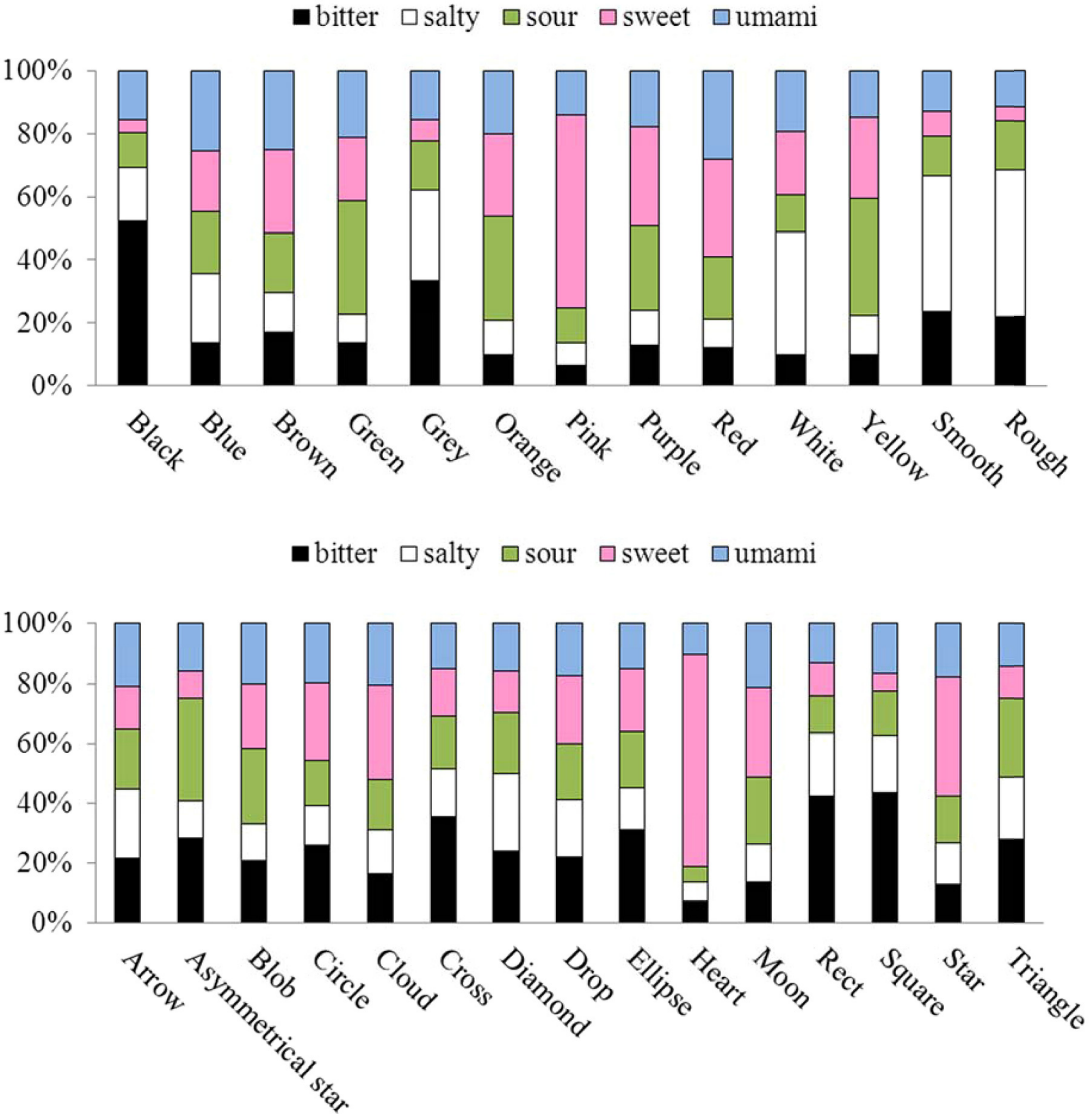
**The taste-patterns for each color (upper panel), each shape (lower panel), and each texture patch (on the right of the upper panel).** Note that the percentage of the bitter, salty, sour, sweet, and umami taste terms chosen for the visual stimuli are represented by the fill-in colors black, white, green, pink, and blue, respectively.

First, we visualized the associations between each stimulus and the five basic tastes in **Figure 3**, and performed Chi-square tests in order to see whether certain taste(s) were more often associated with one taste than the others. As can be seen in **Table 2**, all the colors except blue were associated with certain taste(s). The results of *post hoc* Chi-square tests revealed that some colors were certainly more strongly associated with one taste than with other tastes, including black with bitter, green with sour, pink with sweet, and white with salty.

**Table 2:** Results of Chi-square tests for each stimulus by all four groups of participants (*N* = 428).

Stimuli	Strongly associated taste (exact count in brackets)	χ^2^ tests for taste-patterns across four groups	χ^2^ for the Country × Taste interaction in log-linear analyses
Black	Bitter (223)*	296.74*	18.14
Blue	–	16.51	36.54*
Brown	–	27.89*	16.24
Green	Sour (155)*	91.35*	72.91*
Gray	–	100.79*	39.27*
Orange	–	81.44*	38.03*
Pink	Sweet (261)*	457.47*	28.59
Purple	–	66.46*	51.87*
Red	–	79.99*	33.74*
White	Salty (166)*	111.60*	68.96*
Yellow	–	108.61*	69.37*
Arrow	–	8.87	13.31
Asymmetrical star	–	99.50*	52.51*
Blob	–	19.64	11.35
Circle	–	32.21*	11.67
Cloud	Sweet (134)*	38.10*	15.47
Cross	Bitter (153)*	67.09*	22.87
Diamond	–	22.63*	24.81
Drop	–	3.85	34.74*
Ellipse	Bitter (133)*	39.83*	18.22
Heart	Sweet (303)*	693.29*	53.18*
Moon	–	42.44*	33.60*
Rectangle	Bitter (181)*	146.60*	14.40
Square	Bitter (187)*	170.76*	22.95
Star	Sweet (170)*	106.93*	49.21*
Triangle	–	48.71*	8.92
Smooth	Salty (185)*	171.56*	40.91*
Rough	Salty (200)*	224.59*	23.09

*Asterisk denotes *p* < 0.05, after Bonferroni’s correction for multiple testing.*

As can be seen in **Table 1** where the results of this study and two previous studies are summarized, some of our results are consistent with those reported by previous research, such as the white-salty associations (O’Mahony, 1983; Koch and Koch, 2003), and green-sour (Koch and Koch, 2003; Tomasik-Krótki and Strojny, 2008). By contrast, the crossmodal associations consistently observed in these three previous studies, i.e., the red-sweet and yellow-sour associations, failed to reach significance in the present study.

What is more, all of the shapes except the Arrow and Drop were associated with certain taste(s). The results of *post hoc* Chi-square tests revealed that some shapes were more strongly associated with one taste than the others, including cross/rectangle/square with bitter, and cloud/heart/star with sweet. The results also revealed that both the smooth and rough texture patches were strongly associated with the salty taste.

**FIGURE 4:**
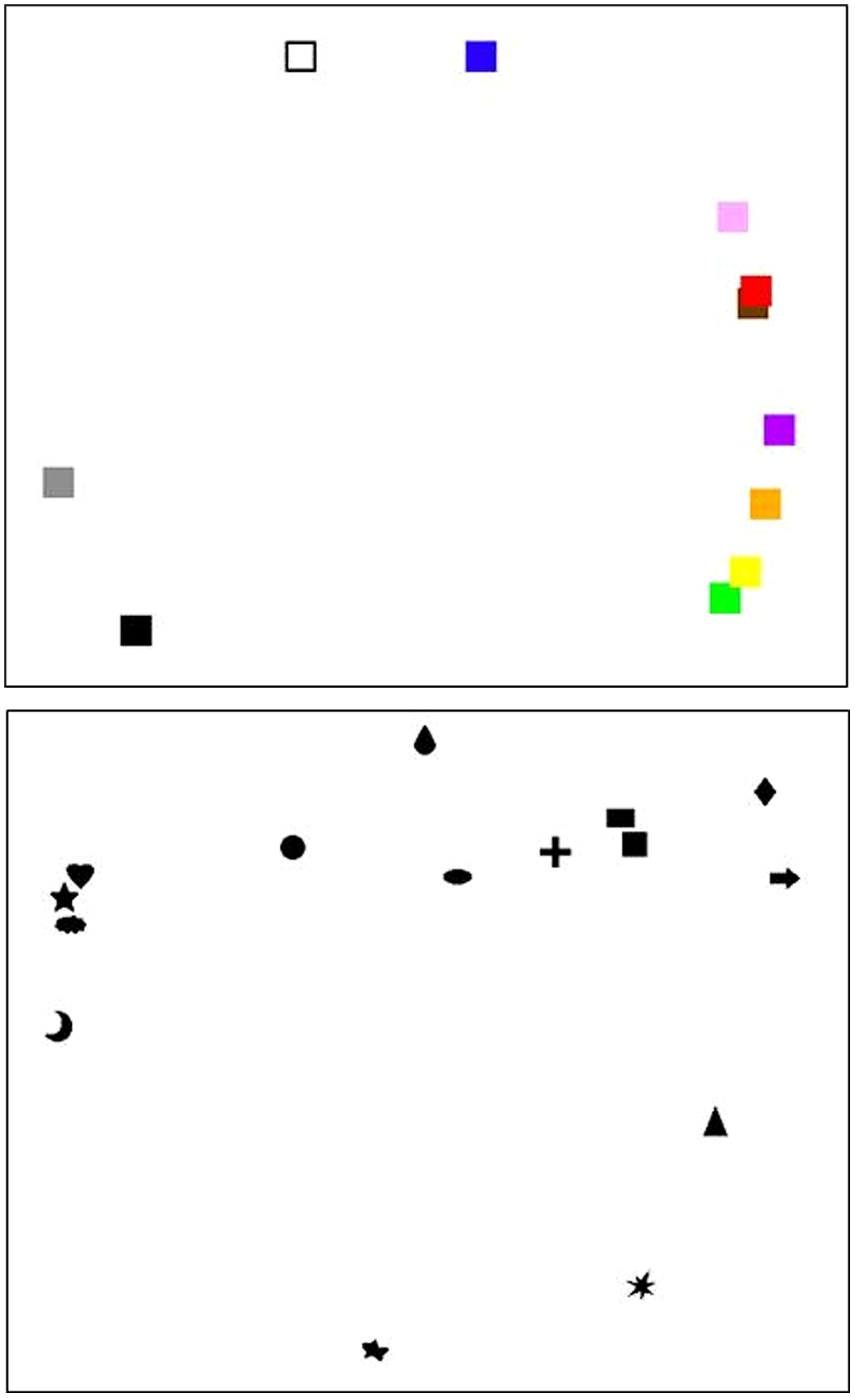
**Multi-dimensional scaling of distances among colors (upper panel) and shapes (lower panel).** The thumbnails of the targets (color patches in the upper panel and shapes in the lower panel) were arranged in the way that their distance between each other reflects their dissimilarity in taste-patterns. Data from all countries combined, weighted by the confidence rating.

After weighting all the data by the participants’ confidence ratings as described in the Methods section, a MDS was calculated for all the colors and for all the shapes, respectively (see **Figure 4**). The results of the MDS are consistent with what was found with the Chi-square tests. This figure clearly shows that black, green, pink, and white are situated far away from each other in the MDS, consistent with the results of the Chi-square tests that they are strongly associated with four different tastes. On the other hand, the MDS figure shows one close cluster comprising the cloud, heart, and stars, and another tightly distributed cluster consisting of the cross, rectangle, and square. These results are consistent with the results of the Chi-square tests that cross, rectangle, and square are associated with bitter, whereas the cloud, heart, and star are associated with sweet instead.

### CROSS-CULTURAL SIMILARITIES AND DIFFERENCES IN THE TASTE-PATTERNS

**FIGURE 5:**
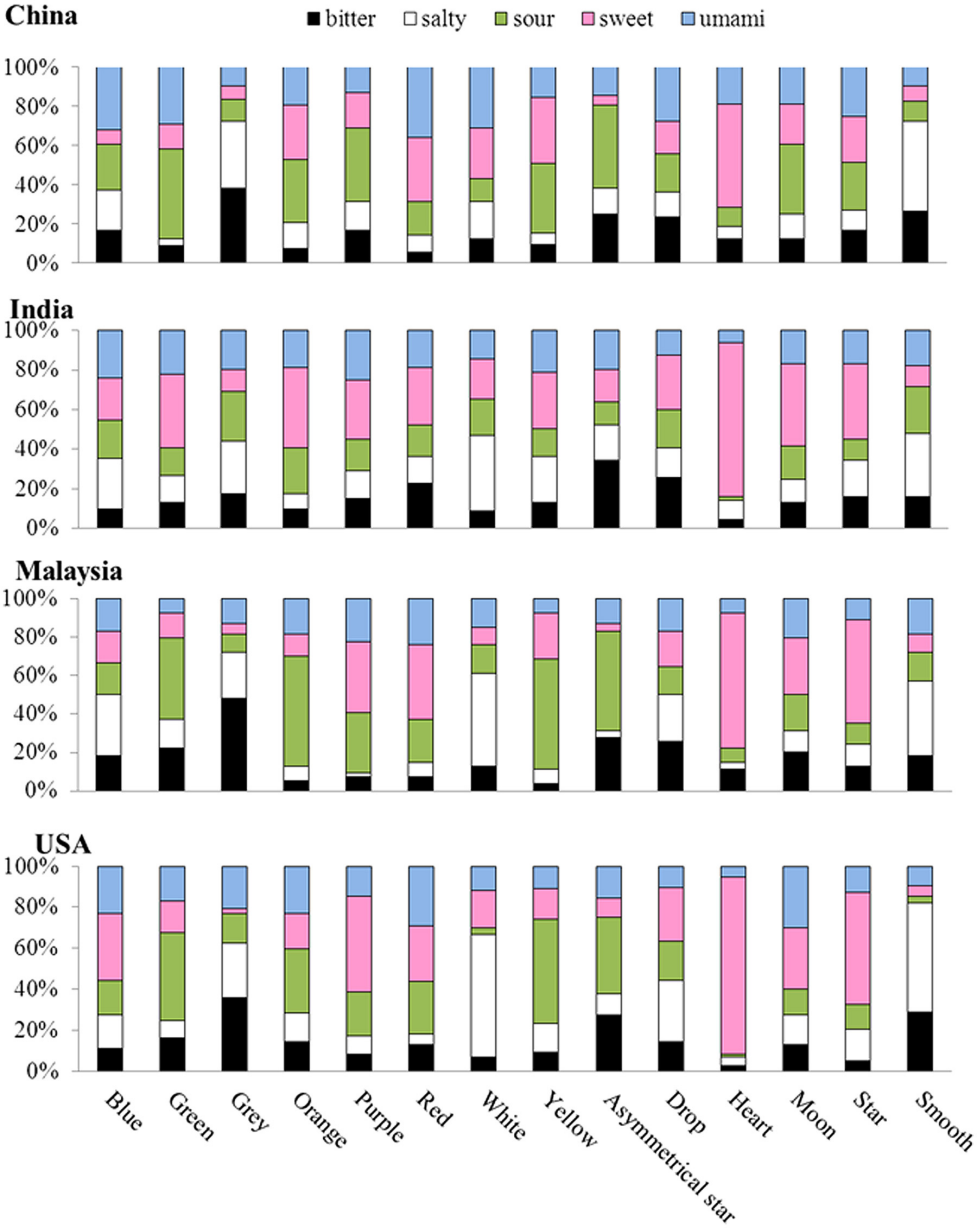
**The taste-patterns for stimuli with which cross-cultural differences were found.** Note that the percentage of the bitter, salty, sour, sweet, and umami taste terms chosen for the visual stimuli are represented by the fill-in colors black, white, green, pink, and blue, respectively.

In order to examine the cross-cultural difference in the taste-patterns, we first performed a log-linear analysis, using Stimulus (28 color-, shape-, and texture-stimuli) × Taste (bitter, salty, sour, sweet, and umami) × Country (China, India, Malaysia, and the USA) as the variables. The final model retained all effects. The likelihood ratio of this model was χ^2^(0) = 0, *p* = 1.00, indicating that the 3-way interaction of Stimulus × Taste × Country was significant, χ^2^ = 860.34, *p* < 0.001. In order to interpret this term, we then performed 2-way log-linear analyses for each stimulus with Taste and Country as variables. As can be seen in **Table 2**, the interaction between Taste and Country was significant for eight of the colors (blue, green, gray, orange, purple, red, white, and yellow), five of the shapes (asymmetrical star, drop, heart, moon, and star), and one of the textures (smooth). The effects of country on the taste-patterns for these stimuli are demonstrated in **Figure 5**.

**FIGURE 6:**
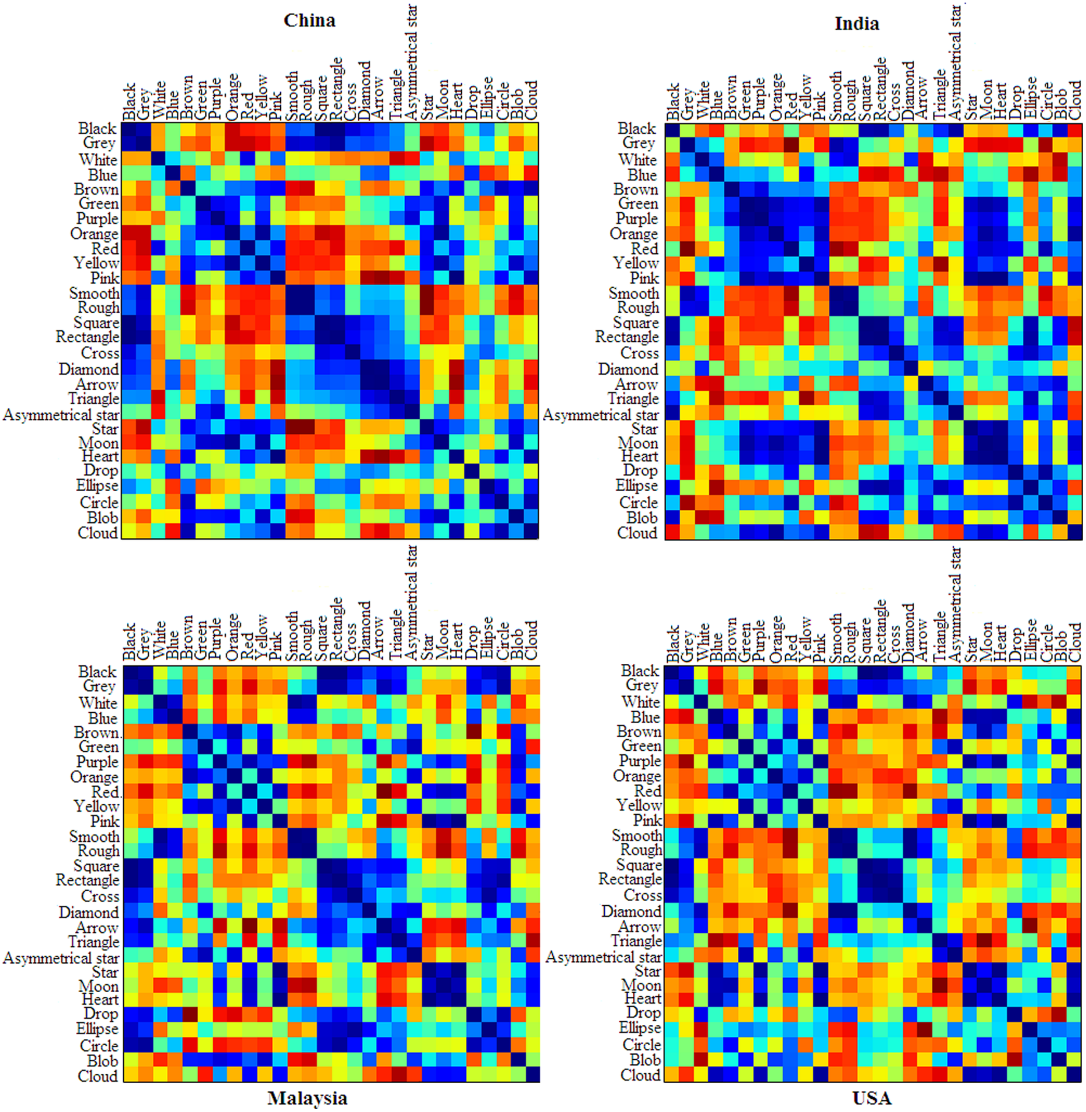
**The RDMs for color-shape-texture-taste associations for each group of participants.** The color of each cell stands for the dissimilarity between the taste-associated patterns of the objects in the respective row and column, with warm colors (more toward the red) denoting high dissimilarity and cold colors (more toward blue) denoting high similarity. That is, the warmer the color of a cell, the more dissimilar the two stimuli are in terms of how they were associated with the tastes. Therefore, the diagonal of each matrix is colored blue to represent the perfect similarity of each stimulus’ taste-pattern with itself.

Representational dissimilarity matrices were also calculated as a function of the culture (see **Figure 6**). First, the blue color displays similar taste-patterns as the color white for the Malaysian participants. The Chinese and Indian groups also showed the same trends; whereas participants from the USA did not. Second, the Indian participants exhibited similar taste-patterns for the colors green, orange, pink, purple, red, and yellow; whereas the Chinese group might further divide these colors into two sub-groups, each one having similar taste taste-patterns, including (1) green, orange, and purple, and (2) red, yellow, and pink. By contrast, the participants from Malaysia and the USA did not show any such pattern of results. By contrast, some cross-cultural similarities can be found in the shape-taste associations across the four cultural groups. That is, they all had a sub-group of shapes with similar taste-patterns, including the square, rectangle, and cross shapes. On the other hand, the Indian, Malaysian, and USA groups all had a sub-group of star, moon, and heart with similar taste-patterns, whereas the Chinese group did not clearly show such a pattern. As for the asymmetrical star shape, drop shape, and rough shape, all the four groups showed different patterns from each other.

These RDMs also allowed us to examine whether any color, shape, and/or texture were associated with the same taste(s). Interestingly, the texture (smooth, rough) has similar taste-patterns with the white color for the participants from India, Malaysia, and the USA, but not for the Chinese participants. What is more, a sub-group of colors (green, purple, orange, red, yellow, and pink) and a sub-group of shapes (star, moon, and hear) had similar taste-patterns for the Indian group, whereas the USA and Malaysian groups also both showed this pattern with the color pink and purple (and only pink for the Chinese group).

**FIGURE 7:**
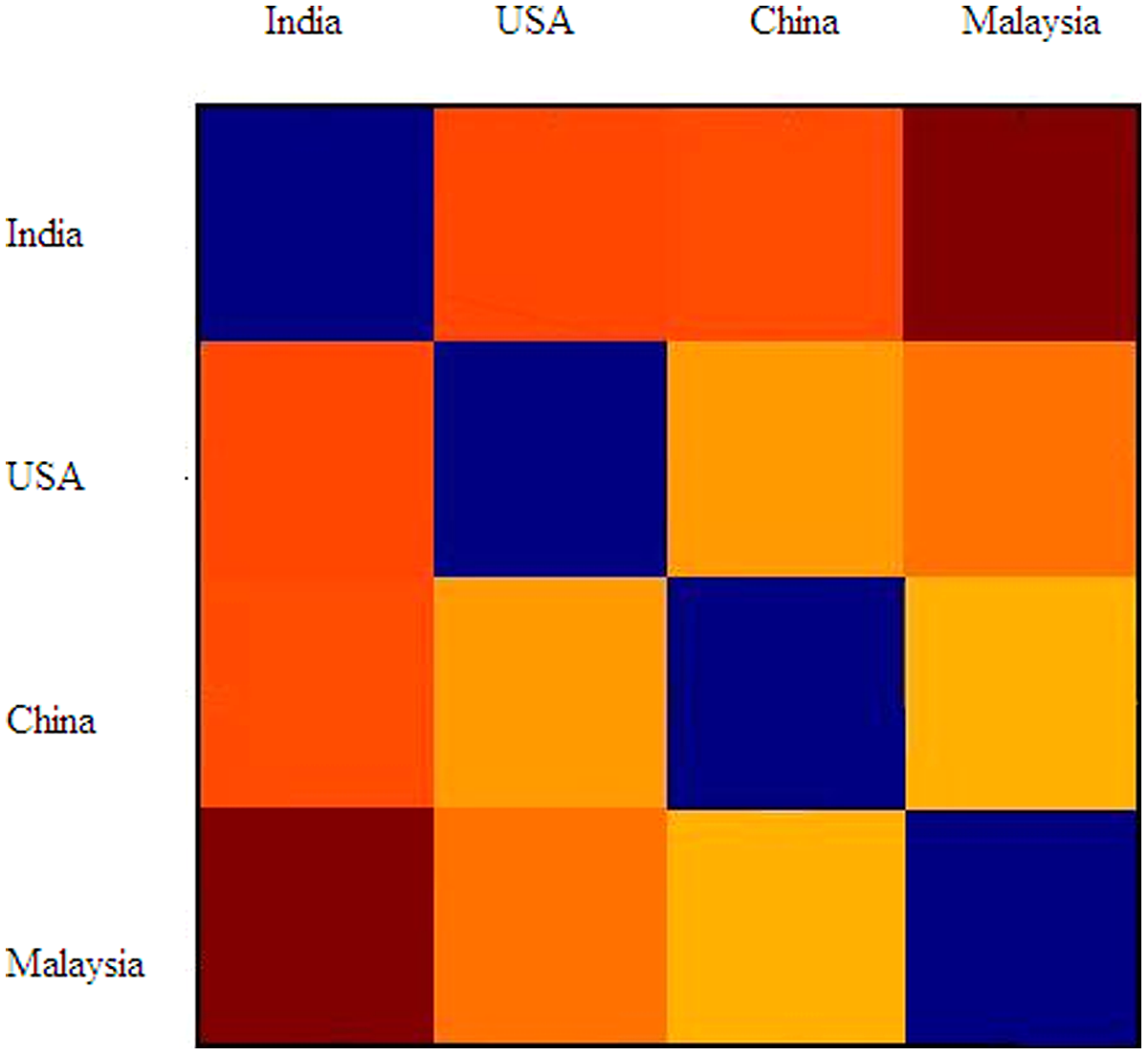
**The cross-cultural RDM.** The color of each cell stands for the dissimilarity between the taste-associated patterns of the culture groups in the respective row and column, with warm colors denoting high dissimilarity and cold colors denoting low dissimilarity. The diagonal of this matrix is still colored blue because of the perfect similarity of each group’s taste-pattern with itself.

**FIGURE 8:**
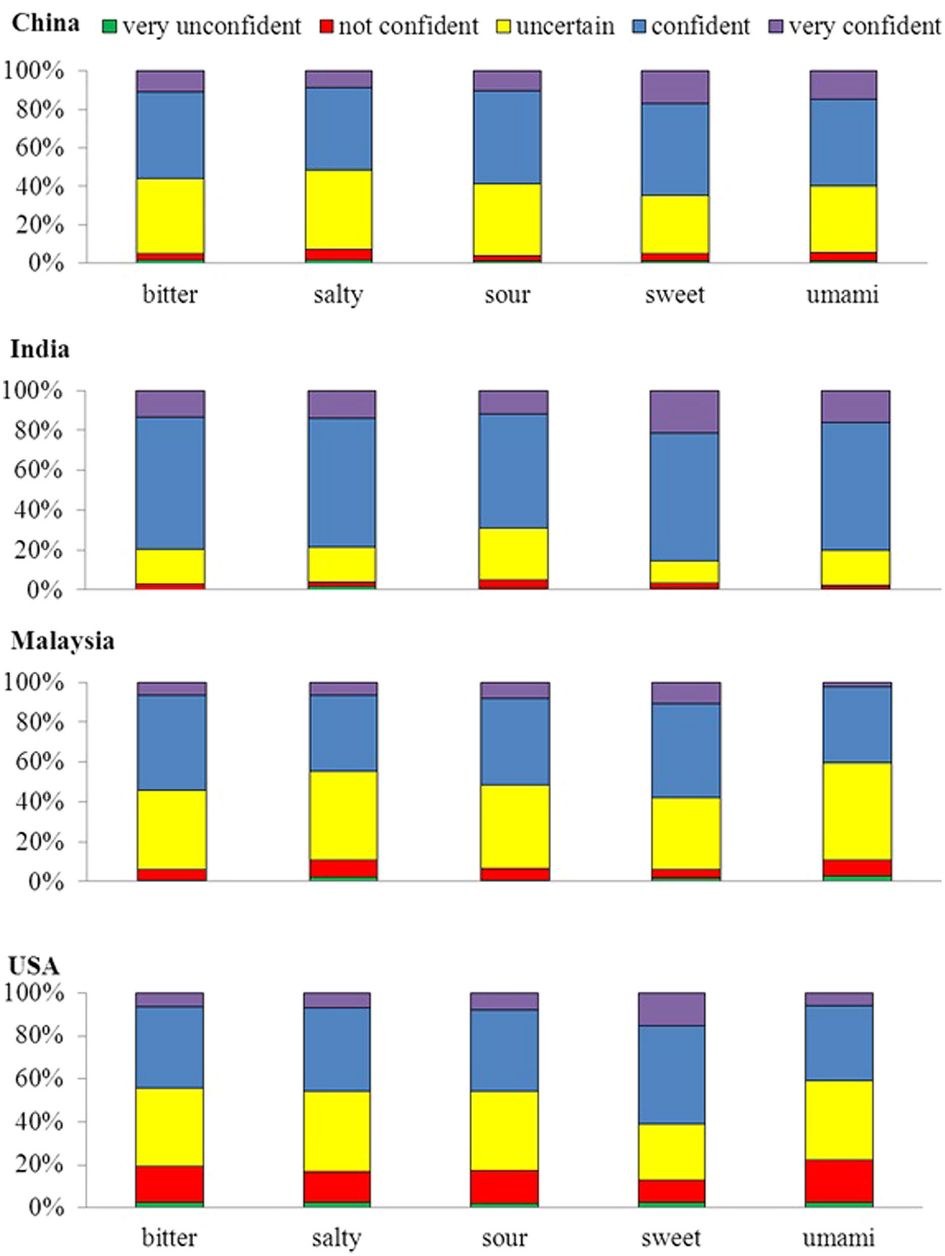
**Confidence ratings for each group of participants.** When choosing a taste term to match the visual stimuli, the participants were also asked to indicate how confident they were that other people would respond in the same fashion as they had. Note that the percentage of the very unconfident, unconfident, uncertain, confident, and very confident chosen for the answer are represented by the fill-in colors purple, blue, yellow, red, and green, respectively.

Last but by no means least, we combined the data from all the groups, and calculated a cross-cultural RDM (see **Figure 7**). Interestingly, this RDM shows that all the countries are approximately equally dissimilar from, or similar to, one another. **Figure 8** also shows each group’s ratings of how confident they were that other people would choose the same taste term for each visual stimulus as they had. Across the four groups of participants, the percentages of those choosing the answers very confident, confident, uncertain, not confident, and very unconfident are 12, 48, 31, 7, and 2%, respectively.

## DISCUSSION

The results of the present study revealed a number of intriguing crossmodal associations between the basic tastes and certain visual features across the four groups of participants from different countries, including (1) the fact that a bitter taste was matched with black, the cross, ellipse, rectangle, and square shapes, (2) the salty taste was matched with the color white, and the smooth and rough textures, (3) the sour taste was matched with the color green, and (4) the sweet taste was matched with the color pink, the cloud, heart, and star shapes. By contrast, the results did not show that the umami taste was strongly associated with any color, shape, or texture in any of the groups of participants. Considering that the umami taste was recognized as one of the basic tastes long after the other four were recognized (Kawamura and Kare, 1987; Mouritsen and Styrbaek, 2014), it is difficult to differentiate whether the participants were simply not familiar with this term, were confused with the usage of this term (namely taste confusion, see O’Mahony et al., 1979), or this taste was just not strongly associated with any one of the visual features presented in this study.

It should be noted that language plays an important role in the matching task, and the participants were asked to make a forced choice. Therefore, the nature of the matching task might elicit more linguistic correspondences than other types of correspondences (see Melara and Marks, 1990; Martino and Marks, 2001; for comparisons between linguistic and non-linguistic correspondences). By contrast, testing with real tastants as opposed to text-descriptors would seem a logical way to avoid this issue in future research. However, it is also possible that some participants would likely still be unfamiliar with the umami taste even when having it in mouth. Importantly, testing with real tastants might be very difficult to do with a large sample of participants from different populations as in the present study. On the other hand, it remains unclear whether the forced choices that the participants made in the present study were the same as the spontaneous and/or automatic responses that they might have made if not given the various options to choose between. It is also possible that the crossmodal associations observed with the matching task is a by-product of the explicit forced choice task (also see Deroy and Valentin, 2011; Velasco et al., submitted), if the participants only chose the least dissonant among unassociated taste words. A future avenue of research might be to use more implicit measures such as the implicit association task (IAT), speeded classification task, and go/no go task.

Comparing to previous studies which either averaged across culture (Tomasik-Krótki and Strojny, 2008) or only tested so-called WEIRD’s (O’Mahony, 1983; Koch and Koch, 2003), our results also revealed some interesting cross-cultural differences in terms of the crossmodal associations that exist between taste and visual features, including eight colors (blue, green, gray, orange, purple, red, white, and yellow), five shapes (asymmetrical star, drop, heart, moon, and star), and one texture (smooth). Compared to the shape-taste associations, it would appear that we have found less color–taste associations that are common across the various cultures (4 out of 11 colors vs. 7 out of 15 shapes), but more cross-cultural differences in the color-taste associations (8 out of 11 colors vs. 5 out of 15 shapes). Peoples’ sensation, perception, and experience of taste/flavor are certainly influenced by their food preferences and consumption, around the latter of which there are obviously dramatic cross-cultural differences (e.g., Kittler and Sucher, 2007). On the other hand, colors convey different esthetic values and meanings in different contexts (Aslam, 2006), and people from different cultures might have very different expectations on seeing the same colored foods (e.g., Shankar et al., 2010; Wan et al., 2014, 2015). Therefore, cross-cultural differences are likely to occur in visual feature-taste/flavor associations, especially in terms of color-taste/flavor associations.

According to the theoretical framework of the categorization of crossmodal correspondences (Spence, 2011), some might be attributable to the same or similar neural coding (i.e., structural correspondences), whereas others might be much more dependent on people’s prior life experience and language, and therefore be expected to potentially vary as a function of culture (i.e., statistical and linguistic correspondences). Therefore, the cross-cultural findings reported here allow us to discuss the nature of crossmodal correspondences in more depth. For example, some strong crossmodal correspondences occur across the four groups, such as the color black/cross shape/ellipse shape with the bitter taste, and pink color/cloud shape with sweet taste. Such correspondences might be structural in origin, and are possibly based to common neural correlates. Alternatively, however, they may also be statistical in nature in that they pick-up on repeated co-exposure of pairs of stimuli or correlated dimensions that people from different cultures have in common. By contrast, those crossmodal correspondences which vary across cultures might be statistical in nature, picking-up on the co-exposure of pairs of stimuli or correlated dimensions that people from different cultures have but others do not, or linguistic correspondences (see also Majid and Burenhult, 2014).

In terms of the whole picture of color-shape-texture-taste associations, we found the four countries, China, India, Malaysia, and the USA, are quite different from each other. In other words, it is difficult to explain these cross-cultural differences simply in terms of the differentiation between Eastern and Western countries. In addition to cultural difference, differences in other domains, such as geographic locations, climates, agriculture, may also play an important role in the formation of crossmodal associations between taste/flavors and visual features (see also Wan et al., 2014, 2015). That being said, it should also be borne in mind that other factors, such as age, gender, travel (which likely means more exposure other cultures), cooking experience, might also vary between participants within a group or cross groups (see also Levitan et al., 2014). It is also possible that, when seeing a taste term, different people might imagine different foods/flavors due to the specific associations they have to each specific taste (see also Majid and Burenhult, 2014). For example, when seeing the word bitter, the Chinese might think of strong tea or traditional Chinese medicine, whereas people from the USA might think of black coffee. The formation of crossmodal associations with taste might be very complicated, and vary from culture to culture, individual to individual.

It should also be noted that two of the shapes in the present study (i.e., named as the “blob” and “asymmetric star”) were very similar to (if not identical to) the round and sharp shapes which were used to elicit the Bouba–Kiki effect (Köhler, 1929; Marks, 1978; Ramachandran and Hubbard, 2001; Bremner et al., 2013). Previous studies have revealed some cross-cultural difference in the crossmodal correspondences between sharp/round shapes and certain tastes/flavors, such as the carbonation of the water (Gallace et al., 2011; Ngo et al., 2012; Bremner et al., 2013) and the bitterness of chocolate (Ngo et al., 2011; Bremner et al., 2013). However, we did not find significant effects of culture on the taste-patterns for these two shapes, which is inconsistent with the findings of previous studies. Martino and Marks (2001) suggested that the crossmodal associations in weak synesthesia are systematic and contextual, whereas those in strong synesthesia are systematic and absolute (see also Marks, 2013; Marks and Mulvenna, 2013).

Therefore, one possible reason for the discrepancy between our results and the results of previous studies might be that these taste-shape correspondences depend to some degree on the particular shapes that are presented and on the total number of shapes presented. For example, it was the marketers of last couple of decades who taught the Western world about the associations between the limpid electric blue and the sweet raspberry taste/flavor (Shankar et al., 2010). A similar discrepancy has also been observed in the literature of odor-shape correspondences. On one hand, Hanson-Vaux et al. (2013) had their participants match 20 odors with a pair of shapes which were often used to elicit the Bouba–Kiki effect. Their results revealed that the lemon and pepper odors were associated with the angular shape, whereas the raspberry and vanilla odors were associated with the round shape, indicating a Bouba–Kiki effect in the odor-shape associations. On the other hand, Seo et al. (2010) asked their participants to match eight odors with 19 irregular shapes (which were referred to as abstract symbols in their paper). Interestingly, the results revealed that pleasant odors were associated with circular or curved shapes, whereas unpleasant shapes were associated with square or angular shapes instead. It is difficult to directly compare the findings in these two studies, as different shapes were used. However, it should be noted that when a large range of varied shapes were used, the more global features (e.g., whether a shape is circular or angular) influences which odors were associated with them. Similarly, when only a blob and an asymmetric star shape, or two categories of round and sharp shapes (see Deroy and Valentin, 2011), were presented, the sharp vs. round difference between them may be signified. By contrast, when a greater number of shapes (15 in the present study) were presented, the sharp vs. round difference might be less salient. That is, when being compared to a lot regular shapes such as a square, rectangle, and so on, the blob and asymmetric star shapes might be classed into one category of irregular shapes. We are currently conducting research to try to understand this issue.

In conclusion, the findings of the present study highlight a number of interesting crossmodal correspondences between four of the tastes (bitter, salty, sour, and sweet) and certain visual features (colors, shapes, and textures). These findings also reveal some cross-cultural similarities and dissimilarities in these correspondences, thus implying the different natures of the different correspondences. That is, some crossmodal correspondences may be more subject to people’s cultural background than others. Future research is needed to test the underlying neural mechanism of the crossmodal correspondences (also see Seo et al., 2010; Chan et al., 2014).

## AUTHOR CONTRIBUTIONS

Each of the listing co-authors made the following contributions to the paper: Charles Spence, Andy T. Woods, Kirsten J. McKenzie, and Xiaoang Wan co-developed the idea for the study and collaboratively designed the study. Andy T. Woods, Xiaoang Wan, and Kirsten J. McKenzie collected the data. Jasper J. F. van den Bosch conducted the data analysis. Jasper J. F. van den Bosch, Andy T. Woods, Xiaoang Wan, Carlos Velasco, and Charles Spence conducted the interpretation of the data. Xiaoang Wan, Charles Spence, Andy T. Woods, Carlos Velasco, and Jasper J. F. van den Bosch drafted the manuscript. All of the authors have read and approved the final version of the manuscript.

## Conflict of Interest Statement

The authors declare that the research was conducted in the absence of any commercial or financial relationships that could be construed as a potential conflict of interest.

## Footnotes

1 Note, though, that texture is not inherently or necessarily visual in nature; it can also be experienced by touching a surface or object. Yet, two types of textures were explored as visual features in the present study.

2 These five tastes terms are often referred to as the “basic tastes,” though it should be noted that there is an ongoing debate as to whether there are basic tastes or not and, if so, how they should be defined (e.g., Delwiche, 1996; Erickson, 2008).

3 The remaining 24 participants came from 13 other countries, including Ireland, Japan, Oman, Romania, Uruguay, Vietnam, Trinidad and Tobago, Sri Lanka, UK, Iran, France, Pakistan, and Korea. Their data were not included in the data analyses of this paper due to the small sample size in each cultural group.

4 The English website for the word umami is http://en.wikipedia.org/wiki/Umami, and the Chinese version is http://zh.wikipedia.org/wiki/%E9%AE%AE%E5%91%B3

5 Note that in this and all following tests where multiple comparisons were conducted, Bonferroni’s correction was used and *p* values are only reported after such correction.
